# Comparative Analyses of MicroRNA Microarrays during Cardiogenesis: Functional Perspectives

**DOI:** 10.3390/microarrays2020081

**Published:** 2013-04-03

**Authors:** Fernando Bonet, Francisco Hernandez-Torres, Franciso J. Esteban, Amelia Aranega, Diego Franco

**Affiliations:** 1 Cardiovascular Research Group, Department of Experimental Biology, University of Jaén, Jaén 23071, Spain; E-Mails: fbonetmartinez@gmail.com (F.B.); fraheto@ujaen.es (F.H.-T.); aaranega@ujaen.es (A.A.); 2 System Biology Group, Department of Experimental Biology, University of Jaén, Jaén 23071, Spain; E-Mail: festeban@ujaen.es

**Keywords:** microRNAs, microarrays, cardiac development, meta-analyses

## Abstract

Cardiovascular development is a complex process in which several transcriptional pathways are operative, providing instructions to the developing cardiomyocytes, while coping with contraction and morphogenetic movements to shape the mature heart. The discovery of microRNAs has added a new layer of complexity to the molecular mechanisms governing the formation of the heart. Discrete genetic ablation of the microRNAs processing enzymes, such as *Dicer* and *Drosha*, has highlighted the functional roles of microRNAs during heart development. Importantly, selective deletion of a single microRNA, miR-1-2, results in an embryonic lethal phenotype in which both morphogenetic, as well as impaired conduction, phenotypes can be observed. In an effort to grasp the variability of microRNA expression during cardiac morphogenesis, we recently reported the dynamic expression profile during ventricular development, highlighting the importance of miR-27 on the regulation of a key cardiac transcription factor, Mef2c. In this review, we compare the microRNA expression profile in distinct models of cardiogenesis, such as ventricular chamber development, induced pluripotent stem cell (iPS)-derived cardiomyocytes and the aging heart. Importantly, out of 486 microRNAs assessed in the developing heart, 11% (55) displayed increased expression, many of which are also differentially expressed in distinct cardiogenetic experimental models, including iPS-derived cardiomyocytes. A review on the functional analyses of these differentially expressed microRNAs will be provided in the context of cardiac development, highlighting the resolution and power of microarrays analyses on the quest to decipher the most relevant microRNAs in the developing, aging and diseased heart.

## 1. Introduction

Cardiac development is a complex process in which multiple cell types are integrated. Two distinct populations of cells contribute to the developing heart, a first heart field (FHF), which mainly leads to the left ventricle, and a second heart field (SHF), which contributes to the rest of the heart [1]. Commitment from the mesodermal lineage into the cardiomyocyte lineage seems to be equally achieved for the FHF and SHF precursors. Both FHF and SHF precursors are mainly dependent on Bmp and Fgf signaling, resulting on the activation of the core cardiac transcriptional machinery, *i.e*., Mef2, Gata, Nkx2.5 and Srf [2]. However, FHF cells are characterized by constitutive expression of Nkx2.5 with only a transient islet-1 expression, whereas SHF cells display strong and constitutive expression of Nkx2.5 and islet-1, providing the first insights into cardiac transcriptional heterogeneity [2]. As development of heart proceeds, distinct cardiomyocyte cell types are established, with distinct cellular, molecular and functional characteristics [3]. Transcriptional regulators, such as T-box, Hands and Pitx2 genes, participate on these processes [4]. In addition to cardiomyocyte formation, the heart develops two additional tissue layers, an inner endothelial lining (endocardium) and external epicardial layer (epicardium). Contribution to the formed heart from these two layers is complex; at discrete regions, the endocardium leads to an epithelial-to-mesenchymal transformation (EMT) configuring the endocardial cushions [5,6], whereas the epicardium displays also a similar EMT transformation, intermingling across the cardiomyocytes and contributing to the formation of the interstitial cells and the coronary vasculature [7,8]. Understanding of the transcriptional control of EMT in both the endocardium and epicardium is emerging, with key roles for Ets, Wt-1 and Snail/Slug transcription factors. In addition to the role of transcriptional regulation, a new layer of complexity is emerging, as non-coding RNAs are also capable of playing a pivotal role during cardiogenesis [9]. microRNAs are non-coding RNA with an average length of 22–24 nucleotides, which are capable of interacting with the 3' untranslated region of coding RNAs (mRNAs), leading to blockage of protein translation and/or mRNA degradation [10]. Understanding microRNA biogenesis has been achieved at a quick pace; however, knowledge about the tissue distribution and functional consequences remains more elusive. However, there is an increasing body of evidence that suggests a highly relevant role of microRNAs in multiple aspects of cardiac development and disease.

## 2. Experimental Section

MicroRNA expression profiles were downloaded from Gene Expression Omnibus [11] GSE32935 (aging heart; [12] Exiqon Mouse microRNA v11.0) and GSE35672 (iPS-cardiomyocytes; [13], Illumina Human v2 MicroRNA expression beadchip datasets). microRNA microarray data from ventricular maturation (MirVana v2.0, miRBase version 8.0) were already available to us, as described by Chinchilla *et al.* [14]. To minimize technical (non-biological) variability among arrays, each data group was independently log2 transformed and then normalized using the quantiles normalization function implemented in the Bioconductor limma package [15], with default parameters run in R software [16], and finally, each probe was Z-scored (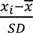). MicroRNA expression data in ventricular development [14], also Z-scored, was identically processed as describe above, but with an initial k-nearest neighbor(KNN) imputation of the densitometry values <1 using the KNN algorithm implemented in the Bioconductor impute package [15] with default parameters. Hierarchical clustering (Euclidean distance and complete linkage), an unsupervised way of grouping samples based only on their gene expression similarities, was carried out using TM4 software suite [17].

## 3. MicroRNAs in Cardiovascular Development

Cardiac development is a complex process in which several cell types are involved. Morphogenetic and transcriptional regulation of gene expression during cardiogenesis has been extensively investigated over the last few decades, providing a good framework to understand the formation of the heart [4]. A novel layer of complexity has been gained by the discovery of microRNAs and their pivotal role in cardiogenesis. Several studies have provided evidence of differential expression of microRNAs during heart development, both during embryogenesis [14], as well as postnatal stages [18], supporting a pivotal roles of microRNA during heart formation. Functional evidence of the role of microRNAs in the developing heart was demonstrated by selective inhibition of *Dicer* in a tissue-restricted manner. Conditional ablation of *Dicer* using Nkx2.5Cre driver mice resulted in embryonic lethality, displaying pericardial edema and cardiac hypoplasia [19]. Inhibition of *Dicer* function using αMHC-Cre mice also resulted in cardiac developmental impairment, and newborns die from heart failure soon after birth [20]. Whereas these studies highlight the importance of microRNA biogenesis for heart development, the role of individual microRNAs in cardiac formation remains largely unknown. However, two examples highlight the importance of microRNAs during cardiogenesis, such as germ-line deletion of miR-1-2, which resulted in ventricular septal defects and early embryonic lethality in a subset of mice [19], and germline deletion of miR-126, which resulted in embryonic lethality due to vascular leakage [21]. Curiously, targeted deletion of other abundantly expressed microRNAs in the developing heart, such as miR-133 or miR-208, have resulted in completely viable mice, suggesting a modulatory rather than a fully determinant role of these microRNAs during cardiogenesis [22,23]. In an effort to grasp the variability of microRNA expression during cardiac morphogenesis, we recently reported the dynamic expression profile during ventricular development [14]. We demonstrate that most of the differentially expressed microRNAs during ventricular maturation (55/486; 11%) displayed increased expression levels, opening a new avenue to explore the role of these microRNAs during cardiogenesis. The rapid pace of deciphering the functional roles of microRNAs has provided a cardiovascular role for 24 of these microRNAs (24/55; ~45%) (Table 1), including multiple validated targets, as nicely compiled in TarBase database [24]. In addition, a large number of microRNA microarrays have been generated in distinct cardiovascular settings, such as human cardiogenesis, embryonic stem cell- and induced pluripotent stem cell (iPS)-derived cardiomyogenesis, as well as the aging and diseased heart, enabling to trace the putative role of these differentially expressed microRNAs in other cardiovascular contexts. Thus, within this review, we will update on the functional role of these previously described differentially expressed microRNAs, and we will explore the putative functional contribution in other cardiovascular settings.

**Table 1:** List of differentially expressed microRNAs during ventricular chamber development [14]. Several of them display a reported cardiovascular role (highlighted in the first column), and several validated targets have been reported, among which those with a cardiovascular role are highlighted. * MicroRNAs reported in humans (hsa-tagged), but not in mice. ** microRNAs reported in mice (mmu-tagged), but not in humans. Note that more that 85% (48/55) of the differentially expressed microRNAs are conserved in both species. iPS, pluripotent stem cell; na, not applicable.

Differentially expressed microRNAs	Cardio-vascular role	Validated targets (cardiovascular role)	Cardiac formation	iPS cardio-myogenesis	Cardiac aging	References
**let-7a**		nras, kras, hmga2	up	up	down	[24]
**let-7b**			up	up	na	
**let-7c**			up	up	na	
**let-7d**			up	up	na	
**let-7i**			up	up	na	
**miR-15b**	miR-15	dmtf1, c22orf5, bcl-2, Chek1	up	down	na	[18,24]
**miR-17**	miR-17	rbl2-p130, ncoa3, e2f1, adkcnna-p21, **fog2**	up	na	na	[24,25,26]
**miR-23b**	miR-23	pou4f2, hes1, **has2**	up	up	na	[24,27]
**miR-24**	miR-24	notch1,mapk14, kiaa0152, dhfr, cdkn2a-p16, alk4, **gata2**, **pak4**, **bcl2**	up	up	na	[24,28,29]
**miR-25**	miR-25	na	up	down	na	
miR-26a			up	up	na	
**miR-30a**	miR-30	**ctgf**, znf294, wnt5a, uap1, tnfrsf10b, tnfaip2, tmem87a, tmem59, tmem41b, tmed7, tmed3, tmed2, tmed10, tmco1, tloc1, ticam2, them4, sypl1, stx7, strn, slc9a3r2, slc9a3r2, slc7a11, slc7a1, slc4a7, slo4a10, slc38a2, slc38a1, slc12a4, sec23a, rpcd1, rbms1, rad23b, rab27b, ptrh1, ptprk, ptgfrn, prpf40a, ppp3r1, ppp3ca, ppp2r4, pgm1, ptprk, ptgfrn, prpf40a, ppp3r1, ppp3ca, ppp2r4, pgm1, pex11b, pafah1b2, pfha2, nufip2, nucb1, nt5e, nt5c3, npr3, np, ncl, napg, myo10, mpdu1, mllt11, mllt1, met, mbnl1, mat2a, lrrc8c, lmnb2, krthb5, kdelc2, jun, itga2, ifrd1, idh1, hnrpm, gpd2, gnai2, gilnt7, galnt1, fxr2, frg1, f2, elmod2, dock7, cpne8, chd1	up	na	na	[30,31,32]
**miR-30d**			up	up	na	
**miR-93**	miR-93	na	up	down	na	
**miR-99a**			up	up	up	
**miR-99b**			up	up	na	
miR-103			up	down	na	
miR-106a			up	down	down	
**miR-122a**	miR-122	trpr6, ndrg3, cd320, bckdk, aldoa, **cck-8**, **caspase-3**	up	na	na	[24,33]
miR-125a	miR-125	Lin28, erbb2, erbb3, zfp385, tor2a, rhebl1, ppt2, mkk7, lin28, jub, entpd4, dus11, ddx19b, arid3b, arid3a, apln, abtb1	up	na	na	[24]
miR-125b			up	up	na	
**miR-126**	miR-126	**vam1**	up	down	na	[24]
miR-130a			up	down	na	
miR-130b			up	down	na	
**miR-133a**	miR-133	**srf**, ptp2, **kcne1**, **hcn2**, **hcn4**, **erg**, casp9, **nfatc4**	up	up	na	[24,34,35,36]
**miR-133b**			up	up	na	
miR-140			up	na	na	
**miR-143**	miR-143	mapk7, mapk12, **aduccine3**	up	up	na	[24,37]
**miR-145**	miR-145	irs-1, flj21308, **dab2**	up	up	na	[24,38]
miR-181a			up	up	na	
miR-181b			up	up	na	
miR-183			up	up	na	
miR-190			up	up	na	
miR-191			up	up	na	
miR-198 *			up	up	na	
miR-202			up	up	na	
**miR-210**	miR-210	efna3	up	up	na	[24]
miR-298			up	na	equal	
miR-320			up	na	na	
miR-322 **	miR-322	na	up	na	up	
miR-324			up	down	na	
miR-351 **			up	na	equal	
miR-373 *			up	down	na	
miR-422b			up	na	na	
miR-453			up	up	na	
miR-455			up	na	na	
**miR-494**	miR-494	na	up	up	down	
miR-494			up	na	na	
miR-503			up	na	down	
miR-513 **			up	na	na	
miR-517 **			up	na	na	
miR-518c			up	na	na	
miR-546 *			up	na	equal	

## 4. A Meta-Analysis of MicroRNA Microarrays in Cardiogenesis and Cardiac Aging

**Figure 1:**
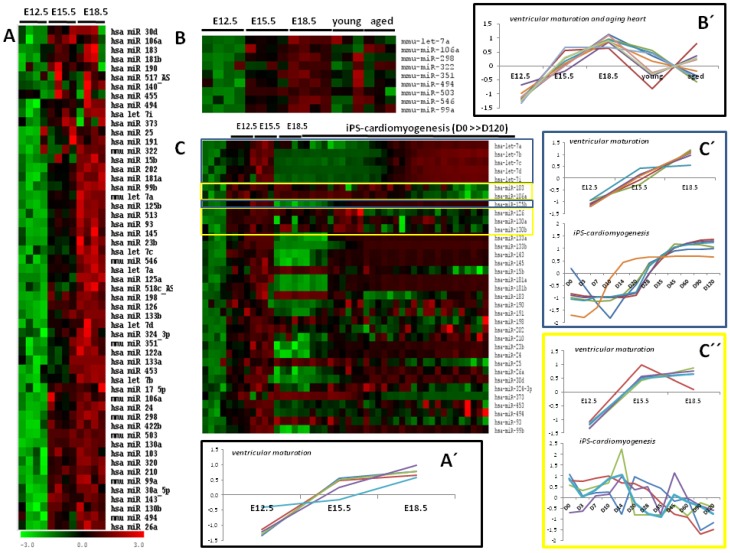
A meta-analyses of microRNA microarrays during in the developing and aging cardiogenesis. Panel **A** illustrates the heatmap of the differentially expressed microRNAs during mouse ventricular development, as described by Chinchilla *et al.* [14]. Panel **B** illustrates the heatmap of the comparative analyses of the differentially expressed microRNAs in the developing and aging mouse heart. Observe that while those differentially expressed during cardiogenesis display increasing expression trends, the young and adult heart display no significant trends. In contrast, comparative analyses of the differentially expressed microRNAs in the developing heart and induced pluripotent stem cell-derived cardiomyocytes, over a maturation period ranging incipient cardiomyocytes (day zero) to 120 days after differentiation, nicely show subset of microRNAs with similar trends (*i.e*., let7 cluster) or opposite trends (see, for example, miR-103 and miR-106b), as illustrated in this heatmap on panel **C**. Panels **A*'***. **B*'***, **C*'*** and **C*''*** are graphical illustrations of microRNA representative expression trends as depicted in heatmaps on panels **A**, **B** and **C**, respectively. Panel **A*'*** displays only a small representation of the full list of microRNAS represented in heatmap of panel **A**. More detailed information can be found in Chinchilla *et al.* [14]. Panel **C*'*** represents a subset of panel **C** microRNAs displaying increased expression levels in both ventricular maturation and iPS-cardiomyogenesis, while panel **C*''*** represents a microRNA subset display increasing expression levels during ventricular maturation, but decreasing expression levels in iPS-cardiomyogenesis.

By using microRNA microarrays, we have recently reported both the most representative differentially expressed microRNAs during ventricular development, as well as the most abundantly expressed [14]. Most recently, using the complementary approach of deep-sequencing, Cao *et al.* [39] have provided similar findings in the developing heart. Interestingly, ten microRNAs were demonstrated to be abundantly expressed; miR-23b, miR-24, miR-23a, miR-375, miR-29a, miR-93, miR-21, miR-25, let-7b and miR-27b, in line with our previous findings. A meta-analyses approach of differentially expressed microRNAs during cardiogenesis comparing the developing mouse heart [14], the induced pluripotent stem cell-cardiomyogenesis [13] and the aging heart [12] reveals interesting patterns, as described below. From 55 microRNAs differentially expressed in the developing heart, 44 were found in the comparison to iPS-cardiogenesis and/or the aging heart. Since the aging heart experiments were performed in mice, only nine *mmu-*microRNA identities were similar, while in the human iPS-cardiomyogenesis, 35 equal *hsa-*microRNA identities were found. Comparison of the differentially expressed microRNAs in the developing heart [14] and aging heart [12] shows that those microRNAs with a progressive increase over time during heart development display arbitrary changes in the adult and aged heart (Figure 1), suggesting that these differentially expressed microRNAs during cardiogenesis are not overtly modified with aging. In line with these findings, only two (miR-494 and let-7a) among those nine microRNAs have been involved in cardiovascular biology, suggesting a rather subtle role in the adult cardiovascular system.

In contrast, comparison of the differentially expressed microRNAs in the developing heart [14] and induced pluripotent stem cells-derived cardiomyocytes [13] revealed 35 shared microRNAs, among which 17 (17/35; 48%) display similar expression profiles during heart development and iPS-cardiogenesis, while seven (7/35; 20%) display an opposite pattern. The remaining 11 microRNAs display arbitrary trends (11/35; 31%). Importantly, 12 out of 17 (70%) with parallel expression profiles have been already involved in cardiovascular biology. Overall, these data nicely illustrate that on the one hand, similar microRNA profiles are observed during cardiac ventricular development and iPS-derived cardiomyogenesis, highlighting the parallelism between the *in vivo* and the *in vitro* system. On the other hand, the fact that the majority of the identified microRNAs in cardiac ventricular and iPS-derived cardiomyogenesis display increasing expression levels with maturation and the fact that a role in cardiovascular development has been established for a large number of them highlights the relevance of microRNA microarray comparison and reinforces the notion of pivotal role for these microRNAs during cardiac development. Furthermore, the extrapolation of these approaches to the diseased heart will further reinforce the significant mean of microarray comparison and will further identify key microRNAs with pivotal roles in cardiovascular development and disease.

## 5. The Cardiovascular Role of Differentially Expressed MicroRNAs during Ventricular Development

Over the last few years, we have witnessed an increasing interest on the contribution of microRNAs to cardiovascular development. Microarray analyses and deep-sequencing studies have increased our knowledge among the microRNA signatures of distinct cardiovascular tissues and conditions. For example, a large number of microRNAs that were initially reported to be upregulated within the developing heart by microarrays [14] have also been reported to be highly expressed in the developing heart by deep sequencing approaches [39]. Vacchi-Suzzi *et al*. [30] have further provided detailed information about the microRNA hallmarks of different cardiac structures, revealing an abundant expression of miR-125, miR-99 and miR-320 in valve tissues and of miR-133 and miR-30 in the myocardium, among those differentially expressed during mouse ventricular maturation [14]. However, the tissue distribution and the functional role of several other microRNAs, such as miR-320, miR-99, miR-125, miR-93 and miR-322 in the developing and/or diseased heart, remains to be elucidated, apart from being highly and dynamically expressed during cardiogenesis [14,30,39], while the function role of others, such as, for example, miR-23, miR-24, miR-83, miR-25, miR-27 and several members of the let-7 family members, is currently emerging, as stated below.

As we have previously mentioned, the heart is a complex structure in which distinct tissue layers and structures can be delineated. Three distinct layers can be delimited, the endocardium, myocardium and epicardium, the myocardium being the most important functionally. In addition, the heart is composed of interstitial tissue, arterial and atrioventricular valves and its own system of blood perfusion, the coronary vasculature. Understanding of the impact of microRNA regulation in these tissues is progressively emerging. To date, the contribution of microRNAs to myocardium biology is emerging at a quick pace. The role of miR-133, together with miR-1, is one of the most extensively studied in both cardiac and skeletal muscle development and disease [40,41]. Chen *et al.* [42] describes the opposite roles of miR-1 and miR-133 in skeletal muscle proliferation and differentiation, despite being transcribed from the same polycistronic unit. Surprisingly, genetic deletion of miR-133a or miR-133b displays no cardiac phenotype [22,23], yet deregulation of miR-133 in distinct cardiovascular diseases has been extensively described, such as during myocardial infarction [43,44] and arrhythmias [45,46]. At the molecular level, miR-133 contributes to repression of cardiac hypertrophy by negatively regulating Nfatc4 signaling [34,35], controlling, thus, the metabolic status of cardiac myocytes [36], and it has been proposed as biomarker in the predicted regression of left ventricular hypertrophy after valve replacement [47]. Most importantly, a significant decrease of miR-133 was observed during zebrafish cardiac regeneration [48], and the experimental modulation of miR-133 by either over-expression or deletion demonstrates a pivotal role of this microRNA in cardiomyocyte proliferation during cardiac regeneration. 

Several other microRNAs, such as miR-143, miR-145 and the miR-17-92 cluster, have also been reported to play a role in cardiac muscle. Miyasaka *et al.* [49] elegantly demonstrate that cardiac development is modulated by hemodynamic inputs controlling miR-143 expression and, thus, cardiac morphogenesis in zebrafish. Knockdown of miR-143 elicits re-expression of retinoic acid signaling components leading to outflow tract and ventricular dysmorphogenesis. Further evidence on the role of miR-143 in heart development has been provided also in zebrafish by Deacon *et al.* [37], who nicely showed that miR-143 targets adducin3, and if impaired, abnormal growth and elongation of the ventricular chambers occurs, leading to decrease cardiac contractility and, eventually, collapse. Interestingly, in the human adult heart, deep-sequencing of the miRNA transcriptome in the left and right atrial chambers has revealed that miR-143 is the highest expressed microRNA in the atrial chambers [50], yet its functional role in the adult (normal and diseased) heart remains to be uncovered.

miR-145 has been primarily implicated in smooth muscle cells, and particularly, it is highly upregulated within the lungs of both experimentally-induced pulmonary arterial hypertension, as well as in patients with idiopathic and hereditable pulmonary arterial hypertension [51]. While there is no evidence of a functional role of miR-145 in the developing cardiovascular development, impaired miR-145 expression has been reported in several cardiovascular diseased conditions, such as acute cardiac infarction and coronary artery disease [52,53]. A functional link between miR-145 and these diseased statuses has been recently reported by Li *et al.* [54], demonstrating that miR-145 protects against oxidative stress-induced apoptosis in cardiomyocytes and also regulating Wnt/beta-catenin signaling through targeting of Dab2 in the ischemic heart [38]. 

The precise contribution of miR-17 remains to be discerned, yet systemic deletion of the miR-17-92 cluster leads to lung hypoplasia and ventricular septal defects at birth [55]. It has been also demonstrated that in embryonic cardiomyocytes, miR-17-92 regulates Fog-2 and, thus, cardiomyocyte proliferation [25], while in neonatal cardiac progenitor cells, it regulates Rb12/p130 [26]. In addition to its role during cardiovascular development, miR-17-92 also plays a pivotal role in the adult heart, since it is differentially expressed in the aging heart [56], and overexpression of this microRNA cluster leads to cardiac hypertrophic cardiomyopathy and arrhythmias [57]. Yet to date, the most described functional phenotype of miR-17 relates to pulmonary hypertension [58,59] and extracellular matrix remodeling [60] in this context.

In addition to the role reported for miR-133, miR-143, miR-145 and the miR-17-92, among those microRNAs differentially expressed during cardiac development, initial hints for miR-494 and miR-210 function in myocyte adaptation and survival during hypoxia/ischemia are also emerging, yet with limited understanding [61,62].

While the myocardium is the most relevant tissue layer in the pumping heart, the role of the interstitial cardiac tissue is progressively emerging, since, in fact, it is the most abundant cell type within the adult heart. In this context, several microRNAs, such as miR-15, miR-30 and miR-24, have been reported to play key functions in the cardiac fibroblasts, yet as previously mentioned, they also seem to provide pivotal roles in other cardiovascular tissues.

miR-15 displays a key role governing cardiomyocyte cell cycle withdrawal and binucleation, by controlling Chek1 expression [63], while in cardiac fibroblasts, it modulates collagen deposition [64]. 

miR-30 display a differential expression in experimental pulmonary hypertension [51]. Within the heart, it is has been reported to play a role in extracellular matrix remodeling, by controlling Ctgf expression, in the context of ventricular hypertrophy [31], a role that might also be linked with downregulation of miR-30 in a model of induced atrial fibrillation with fibrosis and inflammation [46].

Wang *et al.* [65] reported that miR-24 is downregulated after myocardial infarction, correlating with increased extracellular matrix deposition. Forced *in vivo* miR-24 overexpression decreased fibrosis, by controlling furin expression, which in turn, regulated tgf-beta bioavailability. In addition to its role in the interstitial cardiac tissue, a role on the cardiac endothelium has also been demonstrated [28] in the context of myocardial infarction. These authors demonstrate that miR-24 impairs angiogenesis by controlling gata2 and pak4 expression and blocking miR-24 *in vivo* leads to decreased endothelial apoptosis, increased vascularization and preservation of cardiac function in a mouse experimental model of myocardial infarction. Importantly, a role for miR-24 in myocardial cells has also been reported. Li *et al.* [29] reported that ischemia increased miR-24 expression in cardiomyocytes, while forced miR-24 expression in cultured cardiomyocytes increased cell viability and apoptosis, and necrosis rates were reduced. Such effects seemed to be mediated by miR-24 mediated control of the pro-apoptotic gene, Blc2l11. Given these pivotal roles, therapeutic usage of miR-24, in combination with other miRs (miR-21 and miR-221), has provided increased survival and engraftment to cocktail-treated cardiac progenitor cells in a mouse model of coronary artery ligation [66], opening, thus, new therapeutic approaches to heal the diseased heart. 

Apart from microRNAs playing a role in cardiomyocytes and interstitial tissue, two microRNAs have been revealed to play an important role in valve and vascular development. miR-23 has been highlighted in the context of cardiac valve formation. Elegant studies in zebrafish demonstrate that miR-23 controls Has2 expression, therefore regulating the extracellular matrix remodeling during endocardial cushion formation [27]. Additional evidence on the role of miR-23 has been reported in the vascular context, as recently reviewed by Bang *et al.* [67]. Similarly, miR-126 is predominantly expressed in the vascular tissue [21]. Targeted deletion of miR-126 resulted in impaired angiogenesis and vascular integrity [21,68]. In addition, a role for miR-126 in valve development, controlled by VEGF, has been also reported [69]. Yet, more recently, a role of miR-126 has also been ascribed in the developing cardiomyocytes, being regulated by hypoxia and histone deacetylases (HDAC) inhibitors and, therefore, contributing to cardioprotection [70]. 

For other microRNAs, such as miR-122 and let-7, although there is unequivocal evidence of a determinant role during cardiovascular development, it remains to be elucidating at which tissue level they contribute. miR-122 has been reported to play a pivotal role in several endodermal-derived tissues [71,72,73,74], yet its role in the cardiovascular system is just emerging. Huang *et al.* [33] recently reported that miR-122 was upregulated in Pax8 null mutants, which display ventricular septal defects. Analyses of putative targets of miR-122 uncovered that cck-8 and caspase-3 are genuine targets, supporting the notion that Pax8-related cardiovascular defects are mediated by miR-122. Similarly, let-7 has been proposed to play a role in cardiac development [39] and differentiation [75,76], yet full mechanistic insights remain to be clarified. Similarly, abnormal expression of let-7 has been identified in a rat model of myocardial injury (doxorubicin treatment), yet it functional role remains unexplored [77].

## 6. Conclusions & Perspectives

We have reported herein a glimpse of the value that microRNA microarrays can provide to understand the role of microRNAs in cardiovascular development. A simple approach, comparison of the microRNA signature of the developing ventricular chambers, has highlighted that almost 50% of the differentially expressed microRNAs identified have been subsequently shown to play diverse functional roles in the cardiovascular system. Moreover, meta-analyses comparing two additional conditions revealed similar microRNA signatures in the developing cardiac chambers and the differentiating and maturing cardiomyocytes derived from induced pluripotent stem cells, which are not altered in the adult and aging heart. Furthermore, such a proof-of-principle microRNA microarray meta-analyses provide also novel hints, as decoding a subset of microRNAs that behave in the opposite pattern during *in vitro* (iPS-derived) and *in vivo* (chamber maturation) cardiogenesis, opening new avenues to dissect the functional role of these microRNAs in the cardiovascular setting. Moreover, future meta-analyses studies, including not only healthy conditions, but emerging microRNA signatures of diseased status, such as atrial fibrillation, cardiac hypertrophy or ischemia, will certainly increase our understanding of microRNA biology in the normal and diseased heart. In addition, microRNA microarrays also provided novel insights into the intricate biology of microRNA transcriptional regulation and putative target recognition, broadening, thus, the spectrum of their applicability.

As we focus our attention on the developing heart, it is becoming clear that microRNAs play a pivotal role in cardiac muscle biology, the interstitial tissue and also valve development. Yet, one of the challenges that remains ahead of us is to fine-tune our understanding of microRNA function in distinct cardiovascular settings, starting from unravelling their tissue distribution and following with dissecting the role of microRNAs in the epicardium and coronary vasculature. Such endeavors will provide us novel understandings and tools for promising therapeutic usage.
